# Hyperthyroidism is not a significant risk of benign prostatic hyperplasia: A nationwide population-based study

**DOI:** 10.1097/MD.0000000000012459

**Published:** 2018-09-28

**Authors:** Kee-Ming Man, Kuen-Bao Chen, Huey-Yi Chen, Jen-Huai Chiang, Yuan-Chih Su, Samantha S. Man, Dong-Dong Xie, Yi Wang, Zhi-Qiang Zhang, Liang-kuan Bi, Tao Zhang, De-Xin Yu, Wen-Chi Chen

**Affiliations:** a Graduate Institute of Geriatric Medicine, Anhui Medical University; b Department of Urology, The Second Affiliated Hospital of Anhui Medical University, Hefei, Anhui, China; c Department of Medicinal Botanicals and Health Applications, Da Yeh University, Changhua; d Departments of Anesthesiology, Medical Research, Obstetrics and Gynecology, Management Office for Health Data, and Urology, China Medical University Hospital; e Graduate Institute of Integrated Medicine, College of Medicine, Chinese Medicine Research Center, Research Center for Chinese Medicine & Acupuncture, China Medical University, Taichung, Taiwan; f Family Medicine, McMaster University, Hamilton, Ontario, Canada.

**Keywords:** benign prostatic hyperplasia, hyperthyroidism, nationwide population-based study, prostate

## Abstract

Benign prostatic hyperplasia (BPH) is a common disorder in the aging male population. Despite evidence that thyroid status impacts the prostate, the objective of this study was to examine whether patients with hyperthyroidism were at a greater risk for BPH.

This study is a retrospective nationwide population-based cohort study of the Chinese population. Data for this study were retrieved from the Taiwan National Health Insurance Research Database (NHIRD). Overall, 1032 male patients aged 40 years or older with hyperthyroidism diagnosed between 2000 and 2006 were included in the hyperthyroidism group, and 4128 matched controls without hyperthyroidism were included in the non-hyperthyroidism group. Both groups were monitored until the end of 2011. A Cox proportional hazards regression model was used to compute and compare the risk of BPH between study participants with and those without hyperthyroidism.

Patients with hyperthyroidism exhibited a greater incidence of BPH (18.51% vs 15.53%) than did the controls. Furthermore, the hazard ratio (HR) of the hyperthyroidism group was 1.24 times that of the control group [95% confidence interval (95% CI 1.05–1.46)] signifying that there is a significant 24% increase in the risk of BPH with the presence of hyperthyroidism. This increased risk of BPH with hyperthyroidism, however, failed to remain significant (adjusted HR = 1.11, 95% CI = 0.94–1.3) after adjusting for covariates of age (adjusted HR = 2.72, 95% CI = 2.32–3.2), diabetes (adjusted HR = 1.4, 95% CI = 1.17–1.68), hypertension (adjusted HR = 1.74, 95% CI = 1.49–2.03), hyperlipidemia (adjusted HR = 1.25, 95% CI = 1.03–1.53), neurogenic bladder, cystitis (adjusted HR = 1.23, 95% CI = 0.58–2.59), urethral stricture (adjusted HR = 2.01, 95% CI = 0.28–14.47), urethritis (adjusted HR = 1.52, 95% CI = 0.72–3.21), and urinary tract infection (adjusted HR = 1.77, 95% CI = 1.31–2.39).

After adjustment for comorbidities and covariates, hyperthyroidism was not found to be a significant risk factor of BPH in our male study subjects. Further research is warranted to validate our results and elucidate the association of the pathophysiology of these 2 diseases.

## 1 Introduction

The thyroid hormones (THs) triiodothyronine (T3) and prohormone thyroxine (T4) are critical hormones for cell differentiation, growth, and metabolism, and influence the physiological function of nearly all mammalian tissues.^[1–3]^ TH also coordinates short-term and long-term energy needs during metabolic change.^[4]^ Furthermore, THs also influence the development of tumors and proliferation of various types of cancers and metastasis and promote tumor angiogenesis.^[5]^ Hyperthyroidism is defined as having low TSH levels and high T4 levels, and it can also be due to high T3 levels in the presence of normal T4 levels.

Benign prostatic hyperplasia (BPH) is a hormone-dependent disease in aging men. It is a nonmalignant enlargement of the prostate gland and refers to the stromal and glandular epithelial proliferation and hyperplasia that occurs in the transition and periurethral zones of the prostate.^[6]^ The etiology of BPH remains obscure, as it is a multifactorial disease. An early study suggested that abnormal changes of thyroid function and serum levels of TSH are thought to be evidence of the pathogenesis of BPH.^[6]^ In another study, Eldhose et al demonstrated the association between prostate size and TH content and found T3 content to be positively correlated with prostate size, whereas TSH content was negatively correlated with prostate size. No significant association was found between T4 content and prostate size.^[7]^ Another clinical study showed that men with a hypothyroid status might be at a decreased risk of prostate cancer.^[8]^ Evidently, there is a lack of consensus on the effects of hyperthyroidism on BPH risk. Therefore, in the present study, we investigated whether the risk of BPH was increased among a cohort of patients with hyperthyroidism. To do so, a nationwide population-based study design was used, and data were retrieved from the Taiwan National Health Insurance Research Database (NHIRD).

## 2 Methods

### 2.1 Data sources

We designed a cohort study using insurance claim data from the Taiwan National Health Insurance Program. This program has covered 99% of the Taiwanese population since 1995, and is a complete representative sample of the Taiwanese population. For the current study, we obtained longitudinal health insurance databases from NHRI. LHID 2000 contains all the original claim data of 1,000,000 individuals randomly sampled from the 2000 Registry for Beneficiaries (ID) of the NHIRD. These databases contained all medical records including inpatient and outpatient claims from 1997 to 2011. We identified hyperthyroidism patients as hyperthyroidism cohort and patients without diagnosis of hyperthyroidism disease as compared cohort. Diseases were identified using the International Classification of Diseases, Ninth Revision, Clinical Modification (ICD-9-CM). Our study was approved by the Institutional Review Boards and the Research Ethics Committee at the China Medical University Hospital in Taiwan (CMUH104-REC2-115).

### 2.2 Study subjects

**Figure 1:**
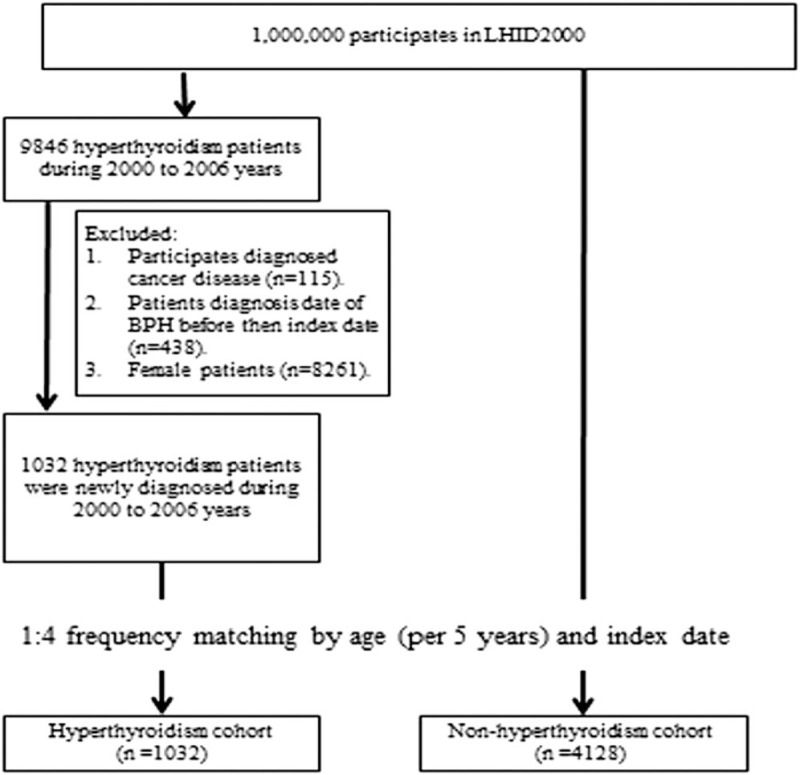
Flowchart shows the enrolment of the participants in the study cohort.

From the medical claims of hyperthyroidism patients, we included 1032 patients diagnosed with hyperthyroidism from 2000 to 2006. Patients with hypothyroidism or other thyroid disorders (including chronic thyroid autoimmune disorder) were excluded from this study (ICD-9-CM: 240, 241, 243, 244, 245, 246) (Fig. 1). The risk of BPH (ICD-9-CM: 600) was computed and compared between study participants with and those without hyperthyroidism. We also compared the lower urological comorbidities between these 2 cohorts. We also excluded the patients whose date of diagnosis of cancer (ICD-9-CM: 140–208) was before the index date. The comparison cohort randomly selected from the population without thyroid gland disorders consisted of 4128 adult males (age ≥40 years) without a history of hyperthyroidism (ICD-9-CM: 242). The sample size of the control group was 4-fold that of the hyperthyroidism cohort.

### 2.3 Outcome measures and comorbidities

The primary outcome was new date of BPH diagnosis (ICD-9-CM: 600). Participates were follow-up until who incidence event, withdraw the NHI, or death, which one occur first. We calculated the sums of follow-up person-years for both cohorts. In addition to age, we also incorporated the baseline comorbidities for hypertension (ICD-9-CM codes 401–405), diabetes (ICD-9-CM code 250), hyperlipidemia (ICD-9-CM code 272) neurogenic bladder (ICD-9-CM code 596.54), cystitis (ICD-9-CM code 595.0), urethral stricture (ICD-9-CM code 598.9), urethritis (ICD-9-CM code 597.80), and urinary tract infection (ICD-9-CM code 599.0) as potential confounding factors.

### 2.4 Statistical analyses

A Chi-square test and 2-sample *t* tests were used to compare differences in demographic characteristics and comorbidities between the hyperthyroidism and non-hyperthyroidism cohorts. We used the 1:4 frequency matching for hyperthyroidism cohort and without hyperthyroidism cohort.

We used a *P* value to present the difference of distributions of age and comorbidities between the 2 cohorts. When the *P* value was less than .05, the distribution was defined as statistically significant between the 2 cohorts. We used a Kaplan–Meier analysis to calculate and plot the cumulative incidence of BPH for the 2 cohorts, and the difference was tested using a log-rank test. Incidence density rates (per 1000 person-years) of BPH were also calculated. Incidence rate was the new BPH cases divided by the sum of the follow-up person-years during the study period. Cox proportional hazards regression analysis was used to assess the hyperthyroidism cohort to the comparison cohort hazard ratio (HR) of BPH with a 95% confidence interval (95% CI). A multivariable model was used to calculate the adjusted HR after controlling for age and all comorbidities. We managed and analyzed the data using SAS 9.4 software (SAS Institute, Cary, NC), and drew the cumulative incidence curve using R statistical software. Statistical significance was defined at a 2-tailed *P* value less than .05.

## 3 Results

In the current study, 1032 male patients who were diagnosed with hyperthyroidism between 2000 and 2006 were included in the hyperthyroidism group, and 4128 male patients, frequency matched by age and index year of diagnosing hyperthyroidism, were recruited as the non-hyperthyroidism control group.

Demographic characteristic data are summarized in Table 1. The population of both groups consisted of 85.85% 40 to 64-year-old men and 14.15% of men aged 65 years or older. There were no significant differences in age between the hyperthyroidism (mean = 53.06, SD = 10.26) and control groups (mean = 52.70, SD = 10.55). The hyperthyroidism patients were more likely to have comorbid conditions than the non-hyperthyroidism group. These comorbid conditions included diabetes (19.67% vs 12.16%, *P* < .0001), hypertension (38.28% vs 25.58%, *P* < .0001), and hyperlipidemia (17.54% vs 9.98%, *P* < .0001). Further, the incidence of BPH between these 2 cohort groups was statistically different (18.51% vs 15.53%, *P* = .019, see Table 1).

**Table 1:**
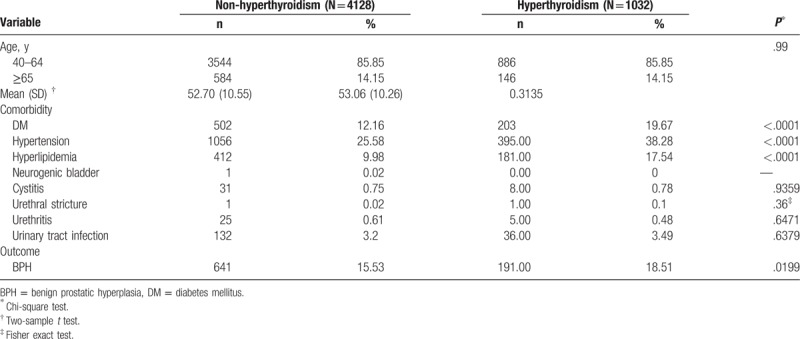
Demographic characteristics and comorbidity in patients with and without hyperthyroidism diseases.

The HR of the hyperthyroidism group is 1.24, 95% CI (1.05–1.46) that of the control group, signifying that there is a significant 24% increase in the risk of BPH with the presence of hyperthyroidism. However, this increased risk of BPH with the presence of hyperthyroidism failed to remain significant after adjusting for covariates including age and comorbid conditions [adjusted HR = 1.11, 95% CI (0.94–1.3)]. This is unsurprising given that separately, age over 65 years (HR = 3.46, 95% CI = 2.98–4.01), and comorbid conditions, including diabetes (HR = 2.18, 95% CI = 1.86–2.57), hypertension (HR = 2.49, 95% CI = 2.17–2.86), hyperlipidemia (HR = 1.77, 95% CI = 1.48–2.13), and urinary tract infection (HR = 2.31, 95% CI = 1.72–3.10) are significant risk factors of BPH (Table 2). The stratified analysis is summarized in Table 3. The incidence rates of BPH were 28.13 per 1000 person-years and 22.71 per 1000 person-years for hyperthyroidism cohort and non-hyperthyroidism cohort group, separately. Stratified by age group, incidence rates were 23.20 and 70.24 per 1000 person-years for 46 to 64 aged group and older than 65 aged group in hyperthyroidism patients. There were higher incidence rates in non-hyperthyroidism patients (17.78 and 62.96 per 1000 person-years for 40–64 aged group and older than 65 aged group, separately). In 40 to 64 aged group, crude HR was 1.31 (95% CI: 1.08–1.58), but there was no significance in multivariate Cox proportional hazard model (HR: 1.09, 95% CI: 0.9–1.33).

**Table 2:**
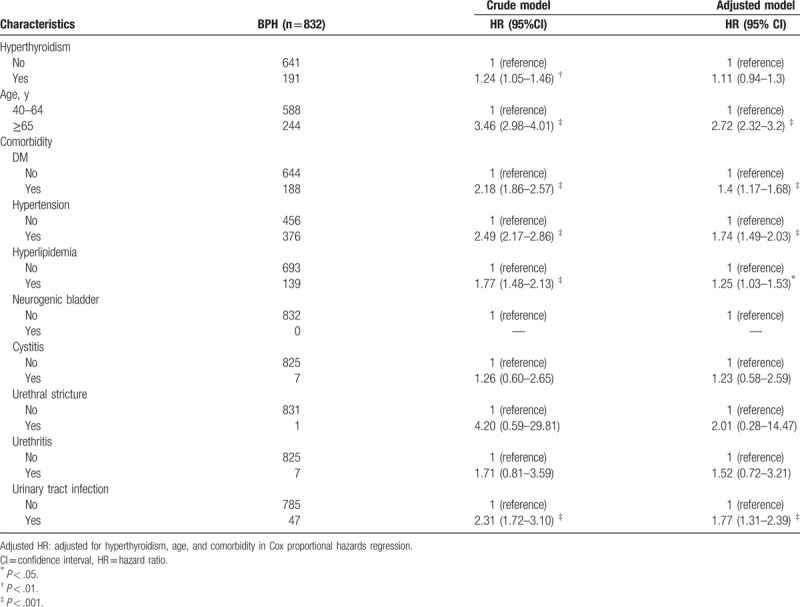
Cox model measured hazard ratio and 95% confidence intervals of BPH associated with and without hyperthyroidism, age, and comorbidity.

**Table 3:**
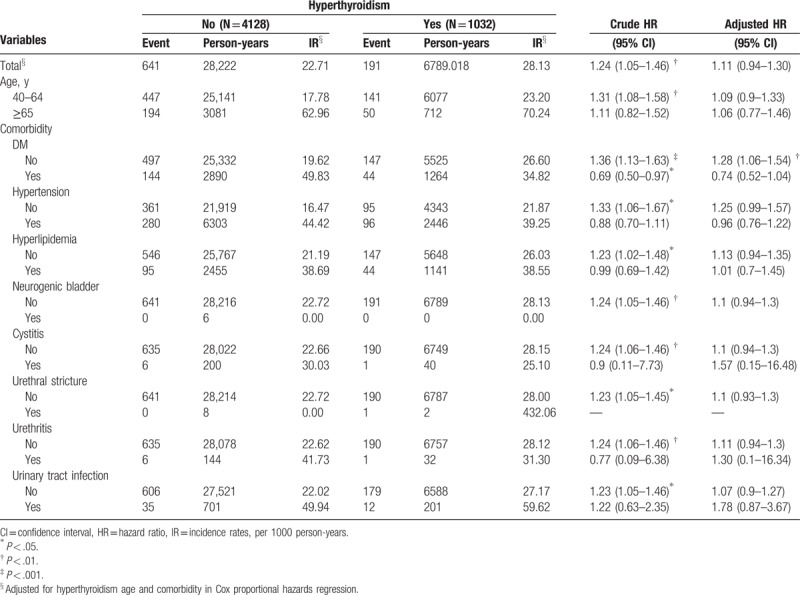
Incidence rates, hazard ratio, and confidence intervals of BPH with and without hyperthyroidism anomaly patients in the stratification by age, DM, hypertension, and hyperlipidemia.

**Figure 2:**
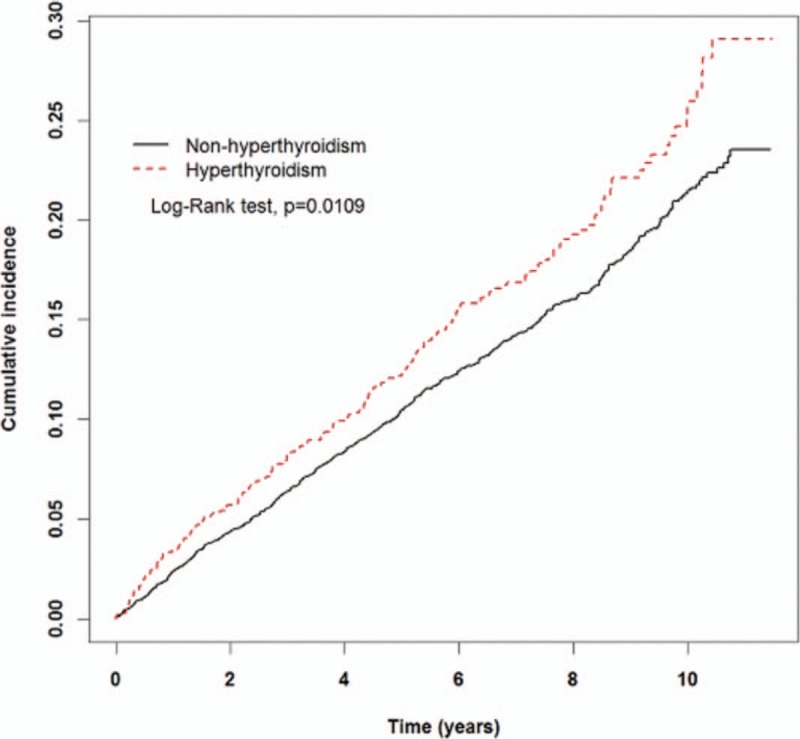
Kaplan–Meier analysis showed that male patients with hyperthyroidism had significantly high rates of developing benign prostatic hyperplasia relative to their comparative cohorts (Log-rank test, *P* = .0109).

Kaplan–Meier analysis showed that male patients with hyperthyroidism had significantly high rates of developing BPH relative to their comparative cohorts (Log-rank test, *P* = .0109, Fig. 2).

## 4 Discussion

In our population-based nationwide cohort study, we analyzed data from 1032 hyperthyroidism patients and 4128 patients selected as a 1-to-4 comparison control cohort. We demonstrated that men with hyperthyroidism and diabetes, hypertension, and hyperlipidemia were at a 124% increase in the risk of BPH in a 5-year follow-up, although there was no significant risk of developing BPH after adjusting for age and the above comorbidity factors.

Several investigators have documented the role of THs in the development of prostate cancer and have demonstrated association of prostate cancer recurrence in patients with high free-T3 levels.^[6]^ In addition, it has been shown that men with hypothyroidism are at a reduced risk for developing prostate cancer compared with euthyroid men.^[9]^ Although several malignancies, such as gastric cancer, thyroid cancer, breast cancer, and ovarian cancer, were established to be associated or not associated with hyperthyroidism and Graves disease, to date, only limited data regarding the association between hyperthyroidism and BPH are available.^[10–13]^

Previous studies have shown that TH contributes to the regulation of BPH and prostate cancer,^[6]^ and that circulating levels of TH determine the size of the prostate.^[14]^ Another study of T3 as a biomarker of prostate disorders indicated that men with BPH or prostate cancer have significantly elevated serum T3 levels.^[15]^ However, T3 showed no effects on normal human prostate cells, or in vitro on TH receptors expressed in a human prostatic epithelium cell line.^[16]^ In another study, the association between prostate size and TH content was assessed. Free T3 was positively correlated with prostate size, while TSH was negatively correlated with prostate size. No significant association was found between free T4 and prostate size.^[7]^

In the real world, there is no evidence that our current cohort study patients with hyperthyroidism are at a higher risk of BPH, although there are several clinical or epidemiologic studies on the relationship between thyroid status and the risk of BPH and/or prostate cancer. Our study is a pilot research project that aims to elucidate a link between BPH and hyperthyroidism in the Chinese population. The specific effect of THs on prostate cells should be clarified further.

In a prospective analysis of α-tocopherol and β-carotene cancer prevention, Caucasian male smokers demonstrated a decreased risk of prostate cancer when in a hypothyroid state, and showed no association between hyperthyroid status and risk of prostate cancer.^[8]^ The Ministry of Health and Welfare of Taiwan released a public health report indicating that the ratio of smokers in the male population of Taiwan was 44.3% in individuals aged 41 to 45 years, and 14.9% in individuals over 66 years of age.^[17]^ In our cohort study, we observed that men with clinical hyperthyroidism were at no significant risk of developing BPH. However, our investigation was limited to only 191 men with BPH, and, as neither smoking habits nor alcohol consumption information was provided by the NHIRD, we were unable to verify if these lifestyle factors contributed to an association between hyperthyroidism and BPH. In addition, epidemiological research has demonstrated that age plays an important role in the risk of BPH, with significant increases in incidence rate over 50% in men above the age of 50, and over 80% in men above the age of 80.^[18]^

Although we retrieved data from the NHIRD, which is highly representative of the general population, several limitations of our study should be addressed. First, a lack of detailed patient information with regard to cigarette smoking habits, body mass index, dietary preference, and family history of systemic disease in the NHIRD may have yielded biased study results because these may be risk factors or comorbidities of BPH. Second, each diagnosis was based on ICD-9-CM codes obtained from administrative data. Detailed information regarding duration and etiology of hyperthyroidism, as well as clinical variables such as TH level during the follow-up period, imaging results, serum prostate-specific antigen levels, and prostate volume were not available. Third, there were no data on measurements and interpretation of THs provided by NHIRD. Thus, hyperthyroidism might be underestimated. Fourth, the follow-up duration is not long for BPH, especially for most of the study population was recruited in their middle ages. Finally, we did not exclude patients with complications of hypothyroidism, long-term L-T4 replacement after anti-hyperthyroid treatment or radioiodine ablation treatment of hyperthyroidism, and the testerone replacement therapy after testical surgery. Factors might remain, leading to bias in our study results.

The evidence suggests that our current cohort study patients with hyperthyroidism are not at a higher risk of BPH, although there are several clinical or epidemiologic studies on the relationship between thyroid status and the risk of BPH. Our study is a pilot research project designed to provide a glimpse into the relationship between BPH and hyperthyroidism in the Chinese population. The direct effect of THs on prostate cells should be further clarified.

## 5 Conclusion

Our cohort study suggests that male patients older than 40 years with hyperthyroidism were not at a greater risk of developing BPH. Further research is warranted to validate our results and elucidate the association of the pathophysiology of these 2 diseases.

## Author contributions

**Conceptualization:** Kee-Ming Man, Huey-Yi Chen.

**Data curation:** Jen-Huai Chiang, Yuan-Chih Su.

**Formal analysis:** Jen-Huai Chiang.

**Funding acquisition:** Wen-Chi Chen.

**Investigation:** Kee-Ming Man, Huey-Yi Chen, Yuan-Chih Su.

**Methodology:** Jen-Huai Chiang, Yuan-Chih Su.

**Project administration:** Wen-Chi Chen, De-Xin Yu.

**Resources:** Kuen-Bao Chen.

**Software:** Jen-Huai Chiang, Yuan-Chih Su.

**Supervision:** Kuen-Bao Chen, Wen-Chi Chen, De-Xin Yu.

**Validation:** Kee-Ming Man, Kuen-Bao Chen, Jen-Huai Chiang, Samantha S. Man, Dong-Dong Xie, Yi Wang, Zhi-Qiang Zhang, Liang-kuan Bi, Tao Zhang, De-Xin Yu.

**Writing – original draft:** Kee-Ming Man.

**Writing – review & editing:** Kuen-Bao Chen, Huey-Yi Chen, Samantha S. Man, Dong-Dong Xie, Yi Wang, Zhi-Qiang Zhang, Liang-kuan Bi, Tao Zhang, Wen-Chi Chen, De-Xin Yu.
